# Quality of Mobile Apps for Child Development Support: Search in App Stores and Content Analysis

**DOI:** 10.2196/38793

**Published:** 2022-11-08

**Authors:** Akeiylah DeWitt, Julie Kientz, Kendra Liljenquist

**Affiliations:** 1 Department of Human-Centered Design and Engineering Seattle, WA United States; 2 Department of Pediatrics University of Washington Seattle, WA United States

**Keywords:** mobile health technologies, early childhood health promotion, child development, parent support technologies, pediatrics, parenting, mobile app, mobile health, mHealth, mobile phone

## Abstract

**Background:**

Following increases in smartphone access, more parents seek parenting advice through internet sources, including blogs, web-based forums, or mobile apps. However, identifying quality apps (ones that respond to the diverse experiences of families) for guidance on child development can be challenging.

**Objective:**

This review of mobile health apps aimed to document the landscape, design, and content of apps in the United States available to parents as they promote their child’s developmental health.

**Methods:**

To understand the availability and quality of apps for early childhood health promotion, we completed a content analysis of apps in 2 major app stores (Google Play and Apple App stores).

**Results:**

We found that most apps do not provide tailored experiences to parents, including cultural considerations, and instead promote generic guidance that may be useful to parents in some contexts. We discuss the need for an evaluative framework to assess apps aimed to support parents on child development topics.

**Conclusions:**

Future work is needed on how to support designers in this area, specifically related to avoiding potential burdens on users and providing culturally informed and equity-driven experiences.

## Introduction

### Background

Intervening early (for children aged 0-5 years) in childhood health has been demonstrated to improve child outcomes [1]. For children born in environments that pose risks to their healthy development (eg, food or housing insecurity), intervening early can offset the degree of impact those risks have on their health outcomes. By enabling parents and caregivers to engage in consistent and evidence-based behaviors that promote their child’s healthy development, more at-risk children will have opportunities to overcome environmental challenges in their development. Children in at-risk environments are less likely to have access to regular pediatric visits [2]. As such, parents and caregivers may need different types of support in being educated about their child’s developmental milestones and engaging their child in activities that support them in meeting those milestones. Parents can find information about developmental milestones through internet searches, from pediatric clinics, at community centers, and other accessible locations [3]. However, translating that information to parenting practices can be difficult and is often exacerbated by ambiguity in how to apply information in limited contexts (eg, in food-insecure environments).

Fortunately, >97% of adults (aged >18 years) in the United States own cell phones with texting capabilities, and 85% of the population in the United States owns smartphones that can download and access apps, with these numbers growing rapidly, particularly for people aged <49 years, who are the most likely the generation to include parents of young children [4]. Researchers have studied the efficacy of phone-based interventions for early childhood health promotion through texting-based programs and mobile apps [5-7]. These apps support parenting practices, including tracking feeding, sleep, and diapers; tracking if a child is meeting essential developmental milestones; facilitating communication with health professionals; finding and implementing health-promoting activities; and collaborating with relevant caregivers. These interventions were designed and tested following guidelines from health and computing fields, with content informed by evidence in the pediatric literature. These apps are also often tested in diverse populations to identify opportunities to promote health equity through design choices [7]. Unfortunately, beyond testing in research contexts, many of these apps are not maintained or deployed to the public because of funding and organizational constraints [8].

Most apps to which parents have access exist in the Apple App and Google Play stores, where app developer experience or qualifications vary widely. These app stores do not have comprehensive guidelines or regulatory oversight for the development of child health apps aside from legal restrictions on claims promising specific health outcomes [9]. App developers may not have access to or knowledge of how to apply design guidelines set by pediatric and human-centered computing researchers. The apps that parents have access to also may not be developed and tested with the same rigor as apps developed in research settings. Although most mobile apps provide a disclaimer that they are not meant to be used to diagnose and thus not directly responsible for health outcomes, they are particularly influential in parenting practice [10,11]. For example, mobile apps can support parents to identify and document patterns in their child’s health that would otherwise go unnoticed and prompt parents to communicate concerning health information to health providers. At the same time, these apps can risk pathologizing health behaviors, raising unfounded concerns, performing self-diagnosis, and causing additional stress in families to micromanage their health. For these reasons, there is a need to critically examine apps aimed to support child development.

In pediatric visit settings, pediatricians sometimes work with parents and caregivers to identify their current resources for child health promotion. These resources can include local community organizations, parent support groups, or access to more immediate communication with health professionals. Pediatricians might also suggest mobile apps to parents to help them organize observations of their child’s development and facilitate collaboration among caregivers. Mobile apps for child development are uniquely positioned to impact multiple areas of parenting experience and child development. By documenting the existing apps available to parents, pediatricians can learn what types of apps parents might be accessing, leading to informed clinical practice when identifying gaps in parenting support. To our knowledge, there has not been any assessment of the quality of these apps to identify how many developers follow evidence-based guidelines in the creation of these mobile apps.

### Related Work

#### Mobile Apps for Child Health Intervention Delivery

Prior work has explored the efficacy of early childhood health interventions administered through mobile systems. Evans et al [6] contributed a pilot evaluation of a texting system that communicated health-related parenting messages to new mothers and measured significant changes in parenting confidence levels. Humphrey et al [12] conducted a feasibility assessment of a mobile app that offered parents feedback on their child’s nutrition and physical activity levels. In this evaluation, they focused on the feasibility of the mobile app specifically for underserved parents and reported both parents’ perceptions of cultural irrelevance in the content and recommendations of the app and dissatisfaction with the quality of the user interface. Wong et al [13] evaluated a mobile app for parent-child collaborative physical activity, reported increased psychosocial wellness for parents and their children, and found the gamified approach for content delivery more effective in improving wellness than the nongamified approach. The content of these mobile health technologies can focus on just 1 aspect of early childhood health (eg, nutrition) or address and support multiple areas of child health (eg, nutrition and sleep). As these are fairly novel technologies, most of these evaluations are limited to documenting if people adhere to these interventions in testing conditions and contribute recommendations for future testing (at larger scales) or design improvements that would improve adherence. Unfortunately, owing to funding constraints, difficulty in coordinating publishing apps, and a lack of incentive for scientists to commercialize their work [8], few of these apps evaluated in academic spaces are published for use in the general public [14,15].

#### Mobile App Design and Regulation in the App Store

Mobile apps present in the public app stores can be developed by both companies and individual developers. Developers are sometimes affiliated with larger companies that partner with health care providers who oversee content and health recommendations. Other developers use their personal experiences to inform the content of their apps [16] or reference published guidelines for health experiences. In the United States, the Food and Drug Administration oversees the development of mobile apps aimed to diagnose and treat any medical conditions [9]. However, oversight into minimal-risk mobile apps, such as those aimed to help patients self-manage their conditions without treatment suggestions or supporting health care providers complete noncomplex tasks, is at the discretion of the Food and Drug Administration.

Both the Apple App and Google Play stores require reviews of mobile apps before reaching the app store. These companies determine the criteria for review, including proof of review processes from external regulatory groups. However, there are several gaps between these processes in assessing the quality and content of the apps. For example, neither of these regulatory processes has requirements for developers to report the sources of the content of their apps, although developers sometimes optionally include their content sources to gain credibility for their app [16]. Developers are also not required to document their design and testing strategies for mobile health apps. For health promotion interventions, researchers recommend extensive engagement with the target population and their environment to inform the content of the intervention [17]. Generally, it is the discretion of the developer to decide when and how the app is modified and when to engage the target population in the design process. Often, developers have multiple feedback mechanisms for future iterations of their apps, including prompts that they build into their app and the app store to engage with user experiences with the mobile app and create plans for updating the app. However, it is important to recognize that many app developers are unable to engage meaningfully with their target populations during the app development process. Instead, developers can refer to guidelines for design and content set by researchers across fields. There is an opportunity to further support developers in generating app content that is responsive to diverse user needs.

#### User Burden in Experiences With Mobile Apps

Mobile apps are uniquely positioned as highly accessible resources with many potential benefits. However, people still sometimes fail to adopt mobile apps with potential benefits or stop using them after a short period, despite having experienced benefits [18]. Often, people may continue to use mobile apps out of necessity while enduring the negative experiences associated with the apps. Suh et al [18] defined this phenomenon as user burden, where computing systems have negative impacts on users. User burden encompasses issues with usability and user experience, as well as burdens defined by Suh et al [18] in their User Burden Scale: difficulty of use, physical, time and social, mental and emotional, privacy, and financial. Suh et al [18] posit that each of these burdens can make it difficult for people to adopt a technology or continue its use. Within health apps, this is particularly important, as the adoption and continued use of mobile apps informs larger scale health outcomes [19]. User Burden Scale has been translated into tangible guidelines for mobile app designers to use [20]. Researchers have also used User Burden Scale to evaluate mobile apps in clinical trials [21] and case studies [22]. In these evaluations, User Burden Scale is posited as particularly useful to address the potential for user burden during the design cycle. User Burden Scale provides a guiding framework to evaluate potential user burdens in mobile app designs.

#### Cultural Competence as an Approach to Health Practice

Cultural competence is commonly defined as an approach to deliver health services that focus on the relevance of culture in health experiences [23]. Cross et al [24] defined cultural competence as supporting changes in health practitioners’ attitudes, health care policies, and practices within the health system. Cultural competence promotes the recognition of how health is affected by diverse cultural experiences and how care practices are more effective when a patient’s health beliefs, values, behaviors, and preferences are emphasized in their interactions with health providers and health systems. Some examples of adaptations to health systems derived from the inclusion of cultural competence include providing interpretation services, partnering with community health workers and traditional healers, and representing diverse populations and experiences using tangible health promotion tools [25]. Cultural competence has been used as a framework to address racial and ethnic disparities in health care [26], highlighting the organizational, structural, and clinical levels as areas of impact. Researchers have also used the cultural competence framework to evaluate the quality of health care delivery in clinical and hospital settings [27].

Researchers in the fields of computing, medicine, and health informatics have identified that health disparities are sometimes worsened by health technologies [28]. Veinot et al [29] identified that technology-generated disparities are pervasive through the adoption, retention, and effectiveness of health technologies. Researchers have used cultural frameworks to improve the design of their mobile apps. For example, the Centers for Disease Control and Prevention (CDC) redesigned their child development app *CDC’s Milestone Tracker App*, to extend the cultural responsiveness of their app to Spanish-speaking families [7]. After evaluating the old version of their mobile app, the CDC found that while the mobile app did offer Spanish translation, the translations were not culturally relevant and thus ineffective for Spanish-speaking families. Their redesign focused on the cultural relevance of translations of contents in the mobile app. Therefore, there is a need for guidance that can support health technology developers as they design and test their systems to respond directly to health disparities and prevent widening them. There is an opportunity to explore the apps of cultural competence as a framework for the evaluation of existing health technologies or as a guide for design and research on health technologies in development.

#### Content Analyses of Mobile Apps

The content analysis method has been used to identify and evaluate mobile apps aimed to address specific health experiences. This method has been used in computing, medical, and health informatics literature to assess mobile health apps in multiple areas. Lukoff et al [30] completed an exploratory review of mindfulness apps and used their findings to engage mindfulness practitioners in conversations about the utility of those apps. Content analysis is also frequently used to evaluate apps related to pregnancy support and postnatal care and in the realm of child development support. Bry et al [31] documented the quality and scope of apps for child and adolescent anxiety and identified the need for apps that use advanced smartphone features and are of higher quality. Mangone et al [32] documented the features and content of apps aimed to support people in pregnancy prevention, highlighting missed opportunities to inform users of helpful information. Yu et al [33] documented the quality of pregnancy and postpartum apps available in both China and the United States by using the content analysis method, finding that many of these apps lacked evidence-based information and functions that supported mental health care. Garland et al [34] designed Psyberguide as another user-friendly resource that supports reviewing and recommending mental health apps. Researchers have also developed and applied evaluation frameworks in their analysis of consumer apps. Meyer et al [35] used the “Four Pillars of Learning” framework to identify opportunities to improve educational apps supported by developmental science. Henson et al [36] developed a framework for evaluating mental health apps, specifically aimed to support patients and clinicians in deciding which apps best support treatment needs. Along that aim, Gordon et al [15] developed an evaluation framework to support the implementation of apps in clinical practice.

To assess the current state of mobile apps for early childhood development and health promotion, we have the following research objectives:

1. What is the landscape of apps that support parents promoting their child’s developmental health, for children aged 0 to 5 years?
2. What aspects of child development support do specific features or design choices address?
3. What burdens are these apps potentially placing on parents or caregivers as they use them?
4. What is the cultural competency of these apps?

## Methods

### App Search and Selection Strategy

We used a content analysis approach based on methodological guidance from Downe-Wamboldt [37] and Mendiola et al [38] to guide the collection and coding of early childhood wellness apps. In January 2022, we searched across Apple (iTunes or App Store) and Android (Google Play) app stores, as identified by Statista [39] as the top 2 most popular app stores in the United States. Our search strings included terms describing child development in simple words (eg, *baby health* and *baby app*). We developed our search terms by combining different strings of terms that are synonymous with *child development app*. The full search strings used in each app store are presented in Multimedia Appendix 1. We limited our search to apps that were available in English and were free to download, as it is recommended that mobile apps for lower-income or disadvantaged communities should be freely accessible [40].

We completed a unique search for each search string in the app stores. We searched for Android apps using the mobile version of the Google Play store, accessed through a web-based smartphone interface. We accessed the Apple apps by searching in the mobile version of the Apple App store. For each of the search result lists, we recorded app titles, respective app stores, and search terms used for all apps yielded from the search. We downloaded all Apple apps to an Apple device running iOS 14 and Android apps to an Android emulator running Android 7.2 on a desktop computer. To mitigate potential biases based on tailored search results, we completed all searches without being logged in to an account on the app stores.

### Selection Criteria

The 3 members of the research team collaborated to develop the inclusion and exclusion criteria for the mobile apps. We included apps if they (1) supported screening or tracking of developmental milestones up to at least the age of 5 years, (2) supported tracking of health promotion behaviors for children up to the age of 5 years (eg, feeding or sleeping), (3) supported English (as the primary language or translations), and (4) were free to download. We excluded apps from the analysis that (1) did not involve baby or child information tracking in some capacity (eg, pregnancy tracking, fertility tracking, or period tracking); (2) only allowed tracking of sentimental mementos; (3) did not offer English translations; (4) were paid apps; or (5) were not downloadable or had restrictions (eg, requiring an early access password).

### Selection Process

We documented the search results on a spreadsheet and flagged duplicates for follow-up across stores. Several apps were present in both app stores but used different names in each app store. A researcher screened the search results in 2 phases by using the inclusion and exclusion criteria. The first phase involved screening the titles of the apps for duplicates between Android and Apple stores and marking apps as potentially relevant. For duplicate apps, we downloaded each and first compared for differences in functionality before excluding a version of the app. In the second phase, we applied the inclusion and exclusion criteria to the app’s descriptions in the app store and confirmed the availability for download. A flow diagram detailing the number of apps present in and after each phase is presented in Section B in Multimedia Appendix 2.

### Data Extraction

A researcher downloaded and reviewed the included apps, documented content into a web-based survey form, and reviewed the data generated on a spreadsheet. This content included (1) the name of the app, app store downloaded from, category in the app store, size in megabytes, highest operating system supported, and latest date of update; (2) the developer name or company, developer’s classification (eg, individual or company), and developer’s self-reported credentials related to early childhood health (if provided in the app posting); (3) privacy permissions that the app requests; (4) in-app purchase content and prices (if offered) and if advertisements are present in the app; (5) other languages offered by apps where English was set as the primary language; and (6) content and delivery structures of the apps, meaning what features each app used (eg, tracking functions or reminders) and what topics were addressed in the apps. We also documented other barriers to accessing mobile apps guided by the literature in health informatics related to mobile health app efficacy for diverse populations, including technical requirements such as internet access, size and data demands of the app, 1-time or subscription costs, and language availability [17].

### Data Analysis

The authors developed codes for the app’s features and content by referencing the national Bright Futures Guidelines for early childhood health promotion [1] and User Burden Scale [20]. With guidance from an author, who is an academic researcher in developmental screening and pediatric health promotion, we reviewed Bright Futures Guidelines and categorized contents by topics covered in well-child visits with pediatricians. From User Burden Scale, we included topics present in the user experience of mobile apps. We have categorized our coding scheme and the peer-reviewed content that informed the coding scheme in Table S1 in Multimedia Appendix 1. We also completed a search of all included apps in January 2022 on Google Scholar to identify if the apps had evaluations published in peer-reviewed venues. An overview of the app characteristics is available in Table S2 in Multimedia Appendix 1.

## Results

### Selection and Inclusion of Mobile Apps

Our initial searches yielded 1348 apps between the Apple App store (574 apps) and Google Play store (774 apps). We excluded 1199 apps during the screening process. We removed 324 (24.1%) duplicates that appeared in both the Apple App and Google Play stores’ search results after comparing functionalities among apps and prioritized including Google Play store versions over the Apple App store versions for the convenience of app review in a web-based emulator. Of the remaining 1024 apps, we excluded 560 (54.7%) apps by title, 400 (39.1%) apps by relevance, and 64 (6.3%) by cost or password-protected download, leaving 149 (39.1%) apps that met the inclusion criteria and were coded. Section B in Multimedia Appendix 2 illustrates the number of apps excluded from the search at each stage of the screening process.

### App Store Characteristics

Table S2 in Multimedia Appendix 1 summarizes the coded app characteristics. In the sample of coded apps, 52 (34.8%) came from the Apple App store and 97 (65.1%) came from the Google Play store. In the Apple App store, 52 apps were distributed across the following categories developed by the Apple App store: Medical (n=28, 54%), Health & Fitness (n=16, 31%), Education (n=5, 10%), Utilities (n=2, 4%), and Lifestyle (n=1, 2%). In the Google Play store, 97 apps were distributed across the following categories developed by the Google Play store: Parenting (n=68, 45%), Medical (n=10, 6%), Health & Fitness (n=8, 5%), Education (n=7, 4%), Books & Reference (n=2, 1%), Lifestyle (n=1, 0.7%), and Tools (n=1, 0.7%).

The earliest operating systems supported ranged from 2010 to 2016 (Google Play store) and from 2014 to 2017 (Apple App store). On average, apps supporting operating systems have been released in the last 7 years. The oldest operating systems were supported by apps from the Google Play store: an app supported phones running operating systems released in 2010. Approximately 38% (37/97) of the apps from the Google Play store supported phones running operating systems released in 2013 or older.

Across both the Google Play and Apple App stores, the dates of the app’s last update ranged from 2014 to 2022. In the Google Play store, the oldest date of the last update was 2015. On average, apps have had at least one update in the last 2 years. Approximately 45.6% (68/149) of the apps were updated in 2022. An app from the Apple App store had not been updated since July 11, 2014, but at the time of writing, it was still available for download from the app store. Unless specified otherwise, the remaining findings are generalized across both the Apple App and Google Play stores.

### App Features

**Figure 1:**
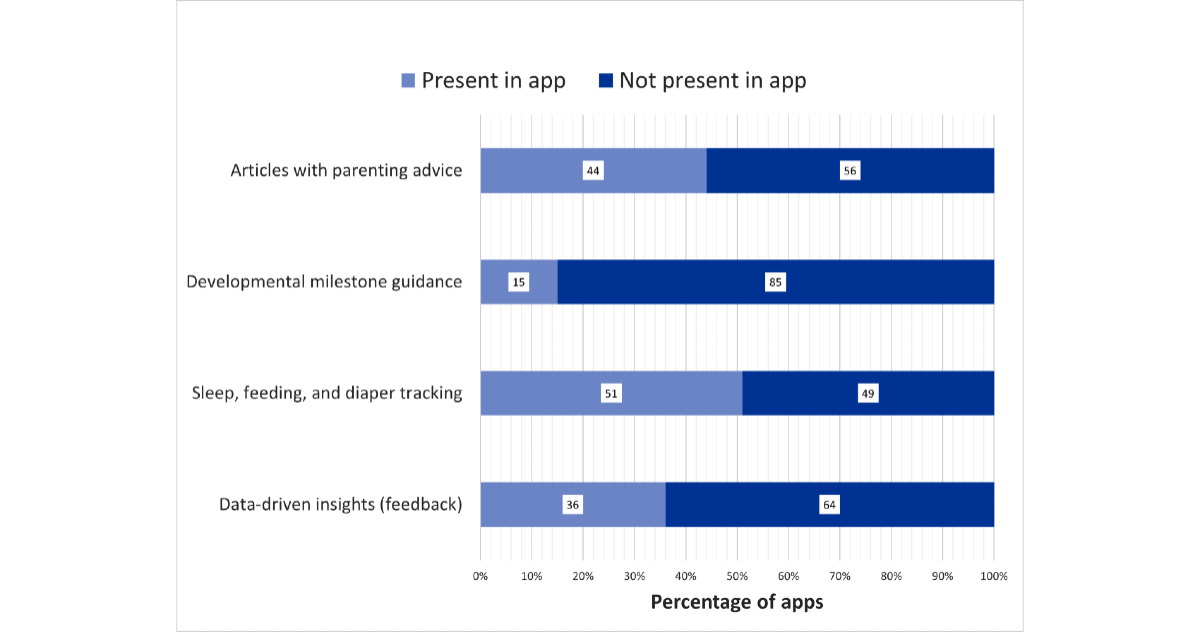
Features present in mobile apps.

We also categorized apps based on those that provided feedback to guide parent action and those that did not provide feedback. This categorization was based on the functionalities related to the user experience for data entry that emerged from the apps during the data-gathering stage. Figure 1 depicts the features present or absent in the mobile apps used in this study.

Apps that provided feedback supported parents in tracking their child’s health data and analyzed the data to recommend that parents pursue specific actions. For example, a parent might use an app to track their child’s milestones, and the app consolidates information (eg, in a summary for parents to review), determines if there is a delay, and recommends the parent contact a pediatrician for a more detailed assessment of their child’s milestone progress. Apps that did not provide feedback allowed parents to track data such as milestones but did not generate personalized feedback on milestones or recommend that parents seek consultation from a pediatrician if their child was delayed in certain milestones. We classified the apps as providing feedback based on their primary and secondary functions offered in the app. Of the 149 apps included in this study, 54 (36.2%) provided feedback to parents. The remaining 63.7% (95/149) of the apps included in this study were classified into the nonfeedback category, as neither their primary nor secondary functions provided feedback informed by personalized information entered by the parent. Textbox 1 highlights some of the main features present across apps that provided feedback to parents and those that did not.

**List of features in included apps.**

**Features that provide feedback to parents (in no specific order)**

- Data visualizations or summaries of user-generated data
- Dynamic checklist of developmental milestones by age (highlighting on track or off track)
- List of development-promoting activities that parents can try
- Screening checklist for specific child health conditions
- Trivia or quiz questions about child health and parenting topics
- Weight, head, and height centile calculator
- Data entry (eg, diapers, feeding or sleep times, words, vaccines, or new teeth) paired with insights and analysis of data
- Growth chart for weight, height, and head circumference that maps and provides guidance about the child’s measurements

**Features that do not provide feedback to parents (in no specific order)**

- In-app articles with parenting guidance
- Sentence-long parenting tips
- Sentimental milestone diary
- Social media forum to connect with other parents
- Videos demonstrating activities
- In-app shopping for baby and parents
- Data entry (eg, diapers, feeding or sleep times, words, vaccines, or new teeth) without insights or analysis of data
- Growth chart for weight, height, and head circumference that does not map or provide guidance about the child’s measurements

### Content and Delivery Methods of Apps

We classified the apps into 2 primary categories. The first category included apps that tracked feeding, sleep, and diaper tracking similar to the tracking recommended for parents immediately following birth. The second category of apps included those that proctored developmental milestone screenings through dynamic questionnaires. In all, 6.7% (10/149) of the apps reviewed in this analysis supported feeding, sleep, diaper tracking, and developmental milestone tracking.

Half (76/149, 51%) of the apps reviewed in this study had a primary function related to feeding, sleep, and diaper tracking. In these apps, parents create a data entry of (1) when their infants fall asleep and for how long; (2) how many diaper changes they have in a day and the quality of the infant’s excretion; and (3) when the infant was fed, for how long, what they were fed with (eg, breastfeeding or bottle), and which breast the breastfeeding parent used during their feeding session. Some apps include advanced features, such as generating charts detailing average sleep duration, feeding duration, or feeding patterns, if multiple methods are used. However, none of the apps in this category offered feedback based on the data entered by parents. For example, to test the functionalities, a researcher made multiple entries in the apps, demonstrating that the infant had not excreted in over 3 days, as national guidelines for infant health recommend contacting a pediatrician if the infant does not excrete for >3 days. None of the apps flagged this pattern as an issue or recommended the parent contact a health professional.

Of these 149 apps, 66 (44.3%) provided secondary functions, such as access to articles with generic information, which were not personalized to the parent or infant’s unique characteristics. These articles included nonspecific parenting advice, information about child developmental milestones, activities to promote children meeting milestones, or photos and video trackers for sentimental child milestones.

Of the 149 apps in total, 23 (15.4%) in this analysis had primary functions related to developmental health promotion and developmental milestone screening. In these apps, parents complete question sets to check their child’s progress toward milestones in the 5 key skills outlined by Bright Futures: gross motor, fine motor, speech and language, cognitive, and social and emotional skills. After completing question sets, the apps generated a summary of milestone progress, sharing if the child was on track to meet milestones, required extra support to meet a milestone, was ahead in their milestones, or was behind on a milestone. On average, these apps supported milestone tracking from birth to the age of 5 years, and apps ranged in support across health promotion themes from birth to the age of 8 years. All apps in this category recommended that parents connect with a pediatrician to follow up on their child’s developmental progress. In all, 4% (2/53) of the apps in this category shared milestone-dependent activities that parents could follow to promote their child’s progress toward milestones; however, these activities were not tailored to unique constraints that families had (eg, safe environment or resources).

Of the 149 apps, 50 (33.6%) offered content related to early childhood health through articles, web-based forums, or growth charts. Apps in this category typically provide information about child health in a noninteractive way, either through lengthy articles or sentence-long trivia facts. An app in this category allowed users to engage with content in a semitailored way, using a chatbot with predetermined chat options, enabling the user to filter information through interactive means.

### User Burden

We coded apps for their perceived user burden based on 6 user burden constructs [20], including the difficulty of use burden, privacy burden, and financial burden. Using these constructs, we coded for user burdens that might deter users from continuing to use the app in a meaningful way. To address time-based burdens, defined by Suh et al [18] as “requires frequent use or a significant amount of time to use,” we documented the time that it took the researchers to complete onboarding tutorials and develop an understanding of how to use the app. We identified that of the 149 apps, 62 (41.6%) required less than a minute to complete onboarding tutorials. In total, 80 (53.6%) apps required <5 minutes to complete onboarding tutorials, whereas 7 (4.7%) apps required >5 minutes to complete the tutorials. In total, 24 (16.1%) apps required >10 minutes for researchers to understand how to use them. However, it is important to note that the research team is not representative of the target population, and as such, these estimates cannot be extended beyond this context.

To address the difficulty of use burdens, we coded for the amount of information presented all at once and whether that information was overwhelming (ie, identifying learning curves). Suh et al [18] define difficulty of use burdens as “The system does not fit with the abilities of the user and is difficult to use. Example systems: i) A photo editing soft-ware package with a steep learning curve; ii) A website that is not compatible with a blind user’s preferred screen reader.” Following this guideline, we documented the presentation of information in the app, and important information about the app’s user experience (eg, key functions or menus) were readily surfaced to the user. A total of 23 (15.4%) apps presented high amounts of information to the user right away, such as long, text-heavy articles about parenting that required long durations of scrolling in the app, highly detailed charts without clear labels, or cluttered home screen or menu items that required the user to click through all of them to understand what they were for. We coded 78 (52.3%) apps that presented large amounts of text without audio or video alternatives, which could present accessibility issues for users with low literacy or vision challenges. We did not directly try out the smartphone’s system accessibility tools in these apps.

More than half of the apps did not require the user to remember extensive information on their own, including the cadence for data entry in apps that require data tracking, key takeaways from guidance on child behaviors and related parenting actions, and returnability for content that may be relevant for the parent later. A total of 136 (91.3%) apps offered functionality within the app that remembered and surfaced information for the user, such as including reminders to track a child’s health metrics or allowing the user to pin relevant pages to access later. We also tracked potential usability concerns related to the mobile app’s system responsiveness, within reliability and user experience. A total of 33 (22.1%) apps posed usability and reliability concerns, including delays in functioning or frequent crashes. These apps also posed additional concerns within the user experience, including requiring repetitive actions to track information (not providing a seamless data-entry experience) or not labeling icons with text descriptions that would require the user to interpret imagery on their own to discern functionality. A total of 6 (4%) apps had color schemes with low contrast between the text and backgrounds. Furthermore, 32 (21.5%) apps had text sizes smaller than 16- to 17-point font, which is not recommended by Google in its Material Design guidelines for developers and Apple’s Human Interface Guidelines.

### Financial Burdens

We also tracked potential financial burdens on the user. Almost half of the mobile apps required in-app purchases to access the full extent of the app’s capabilities or to remove advertisements from the app. Liu et al [41] described the business strategy of these apps as *Freemium*, where apps are free to download but have highly limited functionality without the user paying for premium content. A total of 45 (30.2%) apps required an average 1-time payment of US $8 (SD 11.89), ranging from US $1 to US $60. Furthermore, 25 (16.8%) apps required subscription fees to access the full functionality of the mobile app or remove in-app advertisements. Of those apps, subscriptions averaged to US $57 (SD 48.75) per year, ranging from US $3 to US $225 per year, with an average subscription price of US $23.99 per month. A total of 6 (4%) apps in this analysis included companion tools to supplement app features, which parents would need to purchase to take advantage of the full functionality of the app.

We identified that advertisements were another potentially burdensome feature of some apps. Some advertisements could be bypassed by paying for premium features in the app; as such, advertisements frequently interrupted the user’s experience with the functions of the mobile apps. In total, 3 (2%) apps had advertisement pop-ups that blocked features in the app for at least 20 seconds. Furthermore, 4 (2.7%) apps had advertisements that presented adult content, such as weapons, drugstores, or adult games.

### Privacy and Permissions

The Google Play and Apple App stores have unique systems for tracking the privacy policies of apps, although each store includes information about data-use permissions. Between Apple and Android apps, 30.9% (46/149) of apps listed that data collected from the app would not be linked to the primary user. Among those, 13% (6/46) of apps requested access to potentially sensitive data, such as location, contacts, photos, camera, network connection information (access to internet connection information or Bluetooth devices connected), or existing data on the device. A total of 75 (50.3%) apps requested access to potentially sensitive data such as those outlined earlier but did not provide information on how the data would be used on the download page. For these apps, data-use policies were located directly in the app. Furthermore, of the 149 apps, 40 (26.9%) apps did not provide any information related to privacy policies or data-use permissions and only 12 (8%) apps allowed users to delete their profiles or data collected in the app. All apps requested potentially identifying information, such as the parent’s name and age, child’s name and age, and zip code or approximate location.

### Developers and Credentials

Using information from individual app pages in the app store and external web-based resources (linked from app pages or within the app), broadly, apps were developed by companies; 125 (83.9%) apps were developed by individual associations. Of these apps, 2 (1.6%) were developed by companies in partnership with researchers at a university. We reviewed the company websites posted on app store pages where the app development teams and credentials were listed. Of these associations, only 12 (9.6%) listed subject-matter experts on their app development teams. A total of 7 (5.6%) apps were developed by parents or people who had parented previously. In total, 106 associations did not mention that they included subject-matter experts or parents or caregivers in their development teams. In all, 3 (2.4%) apps were developed by teams from hospitals or medical centers, 2 (1.6%) apps were developed by government agencies, and 1 (0.8%) app was developed by a nonprofit organization. In total, 14 (9.4%) apps were developed by individuals who did not specify their subject-matter expertise or lived parenting experience. An app was developed by 2 parents with an education in sports science. Among the 149 apps, only 13 (8.7%) apps referenced building content in the app following guidelines from government standards (eg, CDC or World Health Organization guidelines) or by citing relevant literature on early childhood health milestones.

### Technical Requirements

We coded technical requirements that may prevent users from continuing to use the mobile app after download. Of the 149 apps, 60 (40.3%) required Wi-Fi or paid cellular data plans to function. In total, 11 (7.4%) apps required more space than specified on the app download page for the downloaded content. Furthermore, 48 (32.2%) apps required an email address to use the full functionality of the app, and 2 (1.3%) apps required a Google account. Of these apps, 4 (8.3%) required a phone number that could receive text messages to sign up for the app.

On average, smartphones made since 2016 hold between 64 and 128 GB of memory storage [42]. On average, operating systems released in 2016 and later require 20 GB of memory to run, leaving between 44 and 108 GB for the smartphone owner’s personal data, including app downloads. For the apps included in this analysis, the average size of the apps across both the Apple App and Google Play stores was 0.0314 GB or approximately 0.07% of the space for a smartphone with only 44 GB of space available. The sizes of the apps ranged from 0.0016 GB (approximately 0.004% of space) to 0.3455 GB (approximately 0.8% of space). For the Apple App store specifically, the average app size was 0.06 GB, while the apps from the Google Play store had a lower average size of 0.02 GB.

### Health Literacy Requirements

We tracked the health and reading literacy [43] levels required by the apps. The content of an app had substantial grammatical problems that hindered the reader’s understanding of the content. We also documented the reading levels required for the content in the apps by selecting samples of reading required for all features in the app. Using the Flesch Reading Ease method, we entered text samples from the apps into a web-based resource that calculated the reading level. In sum, 42.9% (64/149) of the apps in this review presented content below the 7th or 8th grade reading level [44]. Of the 149 apps, 3 (2%) used languages categorized at the college reading level. Of the 113 apps that offered explanations of health topics, 108 (95.8%) apps used simple language (below the 7th or 8th grade reading level) to explain health terms.

### Cultural Competence and Personalization

We also included a dimension of evaluation that focused on cultural competency and tailoring of the apps for diverse groups. A limitation of this work is that we did not include apps developed and presented in primary language aside from English. Mobile apps published in app stores require additional steps to optimize them for globalization or availability across >1 language version of the app store [45]. To access apps with primary languages other than English, a user is required to complete additional steps, including modifying their country or region for their settings across their device, obtaining a virtual private network, or having access to a payment card authorized for use in another country [46]. To represent the search experience of people with limited technology literacy, we retained the default search experience for users operating their devices in the United States.

In this study, only 20.1% (30/149) of the apps included offered languages other than English, including Spanish, Mandarin (Chinese), and German. Although we did not include mobile apps developed in a primary language other than English, we did intend to document other aspects of cultural competency that could be present in the design of mobile apps. In this area, we examined the perceived support of multiple cultural experiences following guidance from the theories of cultural competence, an approach to patient care [47]. We documented the diversity of visual aids in apps that included pictures and videos. Only 12.1% (18/149) of the apps in this study offered images, videos, or icons that depicted people of color. In addition, 24.2% (36/149) of the apps did not offer any personalization features. Of the 75.8% (113/149) of apps that did offer personalization features, those features included changing the name of the child or parent profiles in the app, adding images of a child or family, and changing the colors or themes of the user interface. It is also important to note that several of the apps in this study used gendered language when referring to family configurations (eg, referencing mom and dad, offering only male or female choice for child and parent). An app included in the study, *Baby Sparks—Development App*, offers personalization features that address diverse configurations of families. When getting started in the app, users have the option to self-identify with a broad set of titles, including grandparents, aunt or uncle, development professional, or babysitter. However, similar to the other 12.1% (18/149) of the apps in this study that included diverse imagery, this only includes pictures of families from different races and ethnicities. None of the apps in this study included imagery that presented queer families; caregivers of different ages; or family members with disabilities, different weight ranges, or different religions.

## Discussion

This content analysis found that early childhood health apps support 3 categories of child health monitoring: tracking feeding, development tracking, and learning new information about parenting behaviors. By classifying apps, we documented some of the available apps that can support parents in promoting their child’s healthy growth.

### Searching for Quality Apps

Assessing the quality of mobile apps is an extremely difficult process if the end user is not informed about what qualities they should examine. Parents sometimes seek guidance from trusted sources to navigate the breadth of parenting knowledge available to them, relying on friends and family, curated content from web-based sources, and discussions with web-based communities. Conversations with health providers also inform the decisions that parents make about their parenting practice. Currently, other parents and medical professionals contribute their reviews of mobile apps for child development support on the web. However, reviewing these resources and making an informed decision requires more time and effort from the parents. For this reason, parents generally rely on the content present in the app store to make decisions about which apps are most appropriate for their family’s needs [48].

There is an ongoing discussion on the role of the regulation of mobile apps for health promotion, particularly among apps promoting weight loss and dieting, mental health support, and chronic disease management [9]. Within these areas, it is unclear which groups are responsible for the regulation of content and format for mobile apps [49] and at what level in the app development and publishing process. Mobile apps are positioned to spread information widely and directly impact family actions. For this reason, it is important that mobile apps do not promote inaccurate and potentially harmful information. As mentioned earlier, there are some regulations of mobile apps offered by federal organizations, but the provisions of those regulations can be difficult to interpret for people who are not app developers. However, because the question of regulation in mobile apps is ongoing across business, economics, government, medicine, and design, there is a need to support parents who are actively seeking support from mobile apps and prevent the spread of inaccurate and potentially harmful information to families. As mentioned, mobile app users look toward reviews in the app store for more information about the quality of apps before downloading, but these can sometimes be untrustworthy [50]. As parents seek guidance from trusted sources, there is an opportunity to both develop a framework for the evaluation of mobile apps that parents and pediatricians might rely on when comparing apps in the app store and for designers as they develop child health promotion apps. For example, in both the Google Play and Apple App stores, there are categories (eg, device compatibility, languages offered, and images) that communicate high-level information to users before download. There is an opportunity to leverage how information about apps is presented in the app store (eg, screenshots of app content and descriptions of functionality available in the app), with potential to support end users and people who recommend apps (ie, health providers) as they navigate the available apps in the app store.

Finally, for designers, an evaluation framework can act both as a guide for ethical design outcomes and as a method for evaluating the ethics of apps. In this study, some of the content of our coding framework is directly related to digital ethics (ie, user burden). There is ongoing discussion in computing that references digital ethics and opportunities for digital ethics to act as a guide for design decisions, especially among mobile apps [51]. The Associated Computing Machinery provides a code of ethics [52,53] that designers have previously referenced in their work, to develop useful systems without harming users. Although a review of ethical and unethical practices in mobile app design is beyond the scope of this paper, future work in this area might extend the criteria for the evaluation of mobile apps explored in this paper, supporting designers as they make ethical decisions. For example, the criteria for evaluation might include user burden ratings, technical requirements, areas of child development addressed, cultural competency, health literacy required, and content supported by scientific guidelines. The findings of this study can be used as a foundation for researchers to develop an evaluation framework. Designers and researchers might collaborate in this area to develop a set of criteria that represents both the research and design perspectives and requirements for useful and practical guidelines.

In Table 1 we share a few examples of evaluation criteria that researchers and designers might develop for the evaluation of mobile health apps for child health promotion.

There is also an opportunity to improve the search experience in the app store. For example, compatibility with accessibility features in smartphones can be listed directly in the app store such that the user knows what to expect when downloading an app. The search experience can also be improved by providing search filters; for example, which apps are free and which have advertisements. This information is already available in the app store but cannot be reviewed across multiple apps simplistically (eg, when comparing multiple apps). Another potential barrier in the app store search experience is the prevalence of promoted apps, which are prioritized in the search before other apps, regardless of their quality. This is potentially harmful, as it may mislead users to believing that these apps are of higher quality. Radesky and Hiniker [54] broadly promote platforms (which include app stores) being redesigned to be more child-friendly and suggest that through these design changes, systems will widely be less predatory. Finally, there is a need for future work to examine the readability of privacy statements present in both app stores and mobile apps themselves. Currently, the Google Play and Apple App stores offer high-level summaries of privacy and data-use information, and future work might examine the potential for these summaries to support communicating information related to health data and privacy specifically.

Another adjacent finding worth mentioning is the volume of apps in this study that used a *freemium* business model. App managers have referenced using the *freemium* model to improve the likelihood of users purchasing a premium app after a free trial [41,55], despite lower reviews in the app store. Other researchers have identified that users are willing to pay for apps if they offer more advanced features and improved quality compared with free apps [50]. In this study, the costs of apps ranged significantly, and some app subscriptions were expensive. It is worth considering how lower-income users may be excluded from benefiting from higher-quality apps because of the price burden [20]. Although the use of this business model is at the discretion of companies developing apps and their priorities for app use, there is a need for future work that examines the broad impacts of the *freemium* model for low-income communities and further discussion in industry spaces of the ethics of using *freemium* models for health-promoting mobile apps.

Although beyond the scope of this paper, it is worth noting that several apps included in this analysis were rated as family-friendly but included adult-only content in their advertisements. Other studies have mentioned advertisements in apps that are inappropriate; for example, showing inappropriate advertisement content to children [56]. Although parents are the primary users of the apps examined in this study, future work might address the effectiveness and accuracy of current rating systems for *family* *friendliness* among mobile apps.

**Table 1:** Examples of criteria for the evaluation of mobile apps for child health promotion.

Criteria	Definition	Professionals involved in refining the criteria
Scientific evidence foundation	What are the sources used for health information in the mobile app? Are these sources based on well-founded scientific claims?	Child health researchers, pediatricians, and public health organizations
Areas of child development covered	Does the mobile app address all the areas of child development based on guidance from health authorities?	Child health researchers, pediatricians, and public health organizations
Information communication format	Does the app offer multiple modes of communication (eg, video, audio, text, or pictures)?	Mobile app designers, human-centered computing researchers, and accessibility and inclusion researchers and practitioners
Technical requirements	Does the app require Wi-Fi or data services? Is the app inclusive of devices that are older or have fewer functionalities?	Mobile app designers and human-centered computing researchers
User burdens of the interface	Does the app prevent user burdens on the user as they interact with the app?	Mobile app designers, human-centered computing researchers, and mobile app designers
User burden of access	Does the app prevent cost, health literacy, reading literacy, or security burdens for the user?	Families, public health professionals, health providers, community health workers, and community organizations
Cultural competence	Does the app support a diversity of family experiences by including languages other than English, using nongendered language, presenting diverse family imagery, and offering inclusive health guidance?	Community health workers, community organizations, health providers following culturally informed practices, and diverse families

### Relevancy of Apps for Underserved Groups

Considering the experience of underserved and marginalized people in this space is crucial. Smartphones are widely owned and have the potential to provide new access to information for people without access to care providers or health resources in health networks. We reported space requirements for mobile apps and found that, on average, the size of apps in this category is feasible for the average space available on smartphones. We want to highlight the potential financial burden of these apps. Of the apps reviewed, subscriptions averaged US $57 per year, ranging from US $3 per year to US $225 per year, with an average subscription price of US $23.99 per month. There is a need to further examine the role of financial burdens from apps as a barrier to use by people from lower-income backgrounds in space, as researchers have done for other health apps [57]. Another key finding in this review was related to the lack of culturally diverse visual aids in apps and personalization features. Apps are demonstrated to be more effective when highly tailored to the user’s unique experience [58], and culturally informed approaches to health care discourage using one-size-fits-all approaches to patient care and communication [47]. Finally, the apps included in this review have ≤3 primary features at a time. There is potential for more features in a singular app to burden the user and reduce the likelihood that they will learn all the features present in the app or continue to use the app over a longer period. As such, there is a need for future work that documents the use patterns of parents in this area. To specify, what apps do parents use at different stages of their child’s growth? What is their experience with managing information across multiple apps at a time? Answering these questions may illuminate opportunities for growth in the field when designing new apps for parent support.

### Limitations of This Work

There are several limitations to this work. First, the app market is constantly changing. Since we began this review, it is likely that nearly all of the apps in the study have been updated and improved on. As such, the findings of this study may become obsolete for this domain as apps improve in the future. Another limitation is that we did not assess the compatibility of the built-in accessibility features of these apps. There is a need for future work that examines how these apps respond when features such as screen readers or text magnification are enabled to capture the diversity of experiences for people using smartphones.

Future work may also address the personalization and cultural relevance of experiences in these apps. Tailoring and personalization of care approaches are extremely important in clinical practice, and for apps to be compatible with care happening in clinical contexts, apps should address this need as well. Finally, unlike other content analyses in this field, we did not include app reviews from the app store in the analysis process. This leaves out a key component of information that is usually relevant as users decide what apps to use and engage with other users in the community [59]. Overall, there is a need for more assessments of mobile apps in this area to continue to capture how mobile health apps for child health promotion are changing over time and how they continue to support families.

### Conclusions

We conclude this review by sharing that a plethora of apps are available to parents seeking guidance and support related to their child’s developmental progress. Many of these apps are evidence based, provide tailored feedback, and connect parents with supportive resources outside of their immediate networks. However, these apps are difficult to find within the app store because of the high volume of apps that do not support parents in a meaningful way. In addition, for parents working with their providers to seek mobile apps that work in tandem with clinical care, identifying apps that are high quality and have objectives that meet parent needs can be difficult. There is a need for app stores to promote more apps with evidence-based and inclusive content, accessibility features, and high-quality features. In addition, medical, computing, and health informatics researchers might collaborate to develop an evaluation framework specifically aimed at parents seeking child development support through mobile apps. To respond to systemic changes in health care, researchers and developers may also consider the role of health equity in future evaluations and development of new apps.

## Appendix

Multimedia Appendix 1 Coding scheme and characteristics of included mobile apps.

Multimedia Appendix 2 Search strings and screening process.

## Abbreviations

CDC: Centers for Disease Control and Prevention
