# Supplementary material for: Quality of Mobile Apps for Child Development Support: Search in App Stores and Content Analysis
Source: JMIR Pediatr Parent. 2022 Nov 8;5(4):e38793. doi: 10.2196/38793 (PMC9682452; doi:10.2196/38793)
Supplement: Multimedia Appendix 1 [file pediatrics_v5i4e38793_app1.docx]

## Multimedia Appendix 1

### Table S1 – Coding Scheme

| Code Source | Code | Definition |
| --- | --- | --- |
| User Burden Scale | Requires Help | The app requires help from another person to use it |
|  | Mentally Demanding | The app demands too much mental effort |
|  | Time to Learn | It takes too long to do what you want with the app |
|  | Hard to Learn | The app is hard to learn |
|  | Causes Discomfort | Using the app causes physical discomfort |
|  | Causes Pain | Using the app causes physical pain |
|  | Physically Demanding | Using the app is too physically demanding |
|  | Time Spent | You spend too much time using the app |
|  | Too Often | You use the app more often than you should |
|  | Distracting | The app distracts you from social situations |
|  | Negative Social Impact | Using app has a negative impact on your social life |
|  | Overwhelming Information | The app presents too much information at once |
|  | Feel Bad | Using the app makes you feel like a bad person |
|  | Feel Guilty | You feel guilt when you use the app |
|  | Sharing Concern | You are worried by what information is shared by the app |
|  | Distrust Policy | The app's policies about privacy are not trustworthy |
| Codes generated by the research team | Privacy Work | The app requires me to do a lot to maintain my privacy within it |
|  | Cost | The app is too expensive |
|  | Upfront Cost | The upfront cost of using the app is too high |
|  | Storage Use | How much phone storage is used by the app? |
|  | Languages Offered | What languages are offered in the app? |
|  | Imagery | Images, videos or audio are personally/culturally relevant to me |
|  | Discoverability | The description of the app in the Store does not help me understand it |
|  | Notifications | Notifications are too frequent and do not allow me to customize |
|  | Personalization | Too many/not enough personalization features |
|  | Perceived degree of 'tailored' | App engagements feels tailored to just me |
|  | Multiple children | Allows you to add multiple children |
|  | Tracking multiple children | Tracking multiple children is not difficult |
|  | Health explanations | Explanations of health recommendations are easy for me to understand |
|  | Multiple caregivers | App supports collaboration between multiple caregivers |
|  | Inclusion of caregivers | Doesn't focus exclusively on parent (e g , accommodates non-parent caregivers) |
|  | Inclusion of family diversity | App does not include assumptions about two parent household or uses gendered language |
|  | A lot of reading required | Large amounts of text without audio instructions or alternative media |
|  | Health Literacy | Terms are not overly medicalized |
|  | Reading level of text | What is the average reading level of text in the app? |
|  | Phone data or WiFi required | Does the app require an internet connection to work? |
| Bright Futures | Behavior/Behavior Management | How caregiver(s) address various child behaviors in different settings |
|  | Breastfeeding | Current and future breastfeeding plans and any barriers or contraindications to breastfeeding |
|  | General Guidance on Feeding | Current and future feeding plans related to children who cannot feed themselves |
|  | Car Safety | Existing behaviors to keep the child and other passengers in the car safe |
|  | Care of Teeth and Gums | How parents currently care for the child’s teeth pre, during, and post teething May also ask about dental visits |
|  | Childcare | Care of the child when a primary caregiver is not present |
|  | Excretion | Child’s urination and bowel movements |
|  | Family Planning | Future family planning |
|  | Food and Nutrition | Transitioning to solid foods, appropriate portion sizes and types of foods/drinks the child can have |
|  | Formula Feeding | Current and future formula feeding plans and any barriers or contraindications to formula |
|  | Fussing/Irritability/Crying | How parents soothe the child and identify what the child needs, especially in young infants |
|  | Lab Screening | Lab screening results |
|  | Physical Activity | Healthy levels of physical activity for infants/children |
|  | Reading | Age-appropriate assessment of literacy or parent strategies to promote literacy for their children |
|  | Safety | Behavioral and physical environment safety for the child, excluding water and sleep safety |
|  | Screen Time | Time spent viewing electronic screens |
|  | Sleep | Sleep for the child and caregiver, excluding guidance concerning safe sleep practices for young infants |
|  | Sleep Safety | How the parents keep their the child safe while sleeping |
|  | Toilet Training | How parents address toilet training with their child and/or child's progress toward expected toilet training milestones |
|  | Vaccinations | Child’s vaccinations or vaccination status |
|  | Water Safety | Keeping the child safe in and around water |
|  | Play | Parent's play routines or activities with their child |
|  | Family Routines | Established family routines, or strategies parents currently use incorporate routines with child |
|  | Gross Motor Skills | The gross motor development of the the child |
|  | Fine Motor Skills | The fine motor development of the the child |
|  | Cognitive Skills | Guidance related to the development of cognitive skills |
|  | Expressive Language | Guidance related to the development of language and communication tools that the child uses |
|  | Social/Emotional | The child’s communication, interactions, and emotional reactions to/with peers, family, or others as well as guidance related to developments or changes in mood, temperament, or emotions |
|  | Vision/Hearing | The child’s ability to see, hear and eye/ear health |
|  | Developmental Milestone Surveillance | Developmental milestone/progress surveillance |
|  | Growth Development | The physical growth of the child |
|  | Receptive Language | The development of the child’s understanding of words or language/communication |
|  | Physical Exam | Clinician led physical exams of the child |

### Table S2 - App Characteristics

| Name of App | App Store | Developer | Category | Size (in MB) | Operating systems supported | Primary features in app | Has in-app advertisements? |
| --- | --- | --- | --- | --- | --- | --- | --- |
| Baby Tracker. | Apple App Store | Fitness Labs SRL | Medical | 61.8 | iOS 13.0 or later | Tracking feeding, diaper, sleep | No |
| Babio - Baby Activity Tracker | Apple App Store | Martin Steiner | Health & Fitness | 4.6 | iOS 6.1 or later. | Tracking feeding, diaper, sleep | No |
| baby breastfeeding tracker app - piyolog | Apple App Store | PiyoLog Inc. | Medical | 74.1 | iOS 13.0 or later. | Tracking feeding, diaper, sleep | Yes |
| baby diaries global | Apple App Store | The Baby Diaries Pty Ltd | Health & Fitness | 111 | iOS 9.0 or later. | Tracking feeding, diaper, sleep | No |
| Baby Exercises & Activities | Apple App Store | Aalund Consult I/S | Health & Fitness | 9 | iOS 10.0 or later. | Provides exercises/activities to do with baby | No |
| baby feeding log | Apple App Store | Aron Beaver | Medical | 12.1 | iOS 10.0 or later | Tracking feeding, diaper, sleep | Yes |
| baby journal [babyrepo] | Apple App Store | Permission Inc. | Medical | 85.4 | iOS 11.0 or later. | Tracking feeding, diaper, sleep | Yes |
| Baby Loggy - newborn care log | Apple App Store | Wavescape LLC | Medical | 11.6 | iOS 8.0 or later. | Tracking feeding, diaper, sleep | No |
| Name of App | App Store | Developer | Category | Size (in MB) | Operating systems supported | Primary features in app | Has in-app advertisements? |
| Baby Sparks - Development App | Apple App Store | BabySparks Inc | Education | 149.8 | iOS 9.0 or later. | Development screening | No |
| Baby Steps - Growing Together | Apple App Store | TEDRA SOFT SRL | Health & Fitness | 62.1 | iOS 8.0 or later. | Development screening | No |
| baby time (record & analysis) | Apple App Store | Simfler Inc. | Medical | 169.5 | iOS 11.0 or later. | Tracking feeding, diaper, sleep | Yes |
| Baby Tracker - Newborn Log | Apple App Store | Nighp Softward LLC | Medical | 109.6 | iOS 10.0 or later. | Tracking feeding, diaper, sleep | No |
| baby tracker - nursing helper | Apple App Store | MK App Solutions Korlatolt Felelossegu Tarsasag | Medical | 36.1 | iOS 10.0 or later. | Tracking feeding, diaper, sleep | No |
| Baby Tracker & Breastfeeding | Apple App Store | Wachanga LTD | Medical | 39 | iOS 11.1 or later | Tracking feeding, diaper, sleep | Yes |
| CDC's Milestone Tracker | Apple App Store | Centers for Disease Control and Prevention | Health & Fitness | 58.2 | iOS 10.0 or later | Development screening | No |
| kinedu: baby development plan | Apple App Store | Kinedu | Education | 219.4 | iOS 12.0 or later. | Development screening | Yes |
| Name of App | App Store | Developer | Category | Size (in MB) | Operating systems supported | Primary features in app | Has in-app advertisements? |
| Circle by providence | Apple App Store | Providence health & Services Washington | Health & Fitness | 75.1 | iOS 11.0 or later. | Tracking feeding, diaper, sleep | No |
| Baby Tracker! | Apple App Store | Amila Tech Limited | Medical | 23.7 | iOS 11.0 or later. | Tracking feeding, diaper, sleep | Yes |
| babycare tracker: baby journal | Apple App Store | 倩 赵 (Maxwell Software) | Medical | 29.6 | iOS 11.0 or later. | Tracking feeding, diaper, sleep | Yes |
| BabyDo - Track Your Child's Milestones | Apple App Store | Dr. Jordan Littman, MD | Health & Fitness | 24.5 | iOS 10.0 or later. | Development screening | Yes |
| BabyLine - Baby Nursing | Apple App Store | JeongMin Kang | Medical | 51.5 | iOS 9.0 or later. | Tracking feeding, diaper, sleep | No |
| Bebeto - Baby Development App | Apple App Store | Burak AYTAN | Health & Fitness | 34.8 | iOS 10.0 or later. | Quiz/Trivia about child health | Yes |
| BiliBaby | Apple App Store | Mike Rizzo | Medical | 39 | iOS 10.0 or later. | Screening for Hyperbilirubinemia | Yes |
| Breastfeeding, Pumping Tracker | Apple App Store | LAITKHAUS, OOO Apps | Medical | 65 | iOS 10.0 or later. | Tracking feeding, diaper, sleep | No |
| da baby: newborn tracker app | Apple App Store | Yilei Yang | Medical | 24.3 | iOS 14.0 or later. | Tracking feeding, diaper, sleep | Yes |
| Name of App | App Store | Developer | Category | Size (in MB) | Operating systems supported | Primary features in app | Has in-app advertisements? |
| eat sleep: simple baby tracking | Apple App Store | Make Sail, Inc. | Medical | 7.2 | iOS 6.1 or later. | Tracking feeding, diaper, sleep | Yes |
| Enfamil Family Beginnings | Apple App Store | Reckitt Benckiser Group plc | Health & Fitness | 98.3 | iOS 12.0 or later. | Parenting tips for each week of pregnancy | Yes |
| firstyear - baby feeding timer, sleep, diaper log | Apple App Store | Yi Ding | Medical | 63 | iOS 5.1.1 or later. | Tracking feeding, diaper, sleep | No |
| Growth Charts UK-WHO | Apple App Store | Incubate Ltd/Paediatrics.co.uk | Medical | 7.1 | iOS 8.0 or later. | Centile Calculator for weight, head, height | No |
| Growth: baby & child charts | Apple App Store | Clafou Ltd | Medical | 8.6 | iOS 7.0 or later. | Normal growth monitoring | No |
| Help My Baby Learn | Apple App Store | Help My Baby Learn | Utilities | 21.9 | iOS 11.0 or later. | Development screening | No |
| huckleberry: baby & child | Apple App Store | Huckleberry Labs Inc. | Medical | 42.5 | iOS 11.0 or later. | Tracking feeding, diaper, sleep with specific focus on sleep patterns and consultation from experts | No |
| kidcentric: baby tracker + log | Apple App Store | Kyle Scenna | Medical | 10.4 | iOS 13.0 or later. | Tracking sentimental milestones, keeping track of reminders, generic health reminders (E.g. appointments) | No |
| Name of App | App Store | Developer | Category | Size (in MB) | Operating systems supported | Primary features in app | Has in-app advertisements? |
| Lamaze Play | Apple App Store | TOMY International | Health & Fitness | 345.5 | iOS 9.0 or later. | Scanning app for developmental promotion apps | No |
| Lansinoh Baby App 2.0 | Apple App Store | Lansinoh | Health & Fitness | 112 | iOS 10.0 or later. | Tracking feeding, diaper, sleep, pumping, and parent water consumption | No |
| mammababy - breast feeding app & baby log tracker | Apple App Store | Life'n Stats | Medical | 50.2 | iOS 8.0 or later. | Tracking feeding, diaper, sleep, recording milestones, and tips to support milestones | No |
| my baby - newborn tracker | Apple App Store | Aleksei Neiman | Medical | 30.2 | iOS 11.0 or later. | Tracking feeding, diaper, sleep | No |
| mybaby milestones | Apple App Store | Tamer Abdel -Baset | Utilities | 10.9 | iOS 6.0 or later. | Information about milestones | No |
| n-born - baby feeding tracker | Apple App Store | Ivan Petrashka | Medical | 60.5 | iOS 11.2 or later. | Tracking feeding, diaper, sleep | No |
| Napper: Baby Sleep Tracker | Apple App Store | Napper AB | Lifestyle | 64.3 | iOS 11.0 or later. | Tracking sleep only | No |
| nara baby tracker | Apple App Store | Nara Organics, Inc. | Medical | 21.4 | iOS 11.0 or later. | Tracking feeding, diaper, sleep | No |
| newborn baby tracker & log | Apple App Store | ZENIA, OOO | Medical | 33.4 | iOS 10.0 or later. | Information about activities that promote developmental promotion | No |
| Name of App | App Store | Developer | Category | Size (in MB) | Operating systems supported | Primary features in app | Has in-app advertisements? |
| nursing timer | Apple App Store | Creaplay/Junhyun Cho | Health & Fitness | 16.6 | iOS 8.0 or later. | Tracking feeding, diaper, sleep | Yes |
| Onoco - Baby and Child | Apple App Store | Onoco Limited | Medical | 49.4 | iOS 13.0 or later. | Tracking, feeding, diaper, sleep, developmental milestone tracking, activities for developmental health promotion | No |
| Pathfinder Health | Apple App Store | Samar Parikh | Medical | 34.2 | iOS 10.0 or later. | Development screening | No |
| Saint Francis Baby & Me | Apple App Store | Saint Francis Hospital and Medical Center | Health & Fitness | 23.5 | iOS 12.0 or later. | Resources for early well-child care (includes tracking diaper, feeding) | No |
| Sprout Baby (Baby Tracker) | Apple App Store | Med ART Studios LLC | Health & Fitness | 53.9 | iOS 13.0 or later. | Tracking feeding, diaper, sleep and monthly developmental milestones, information about general parenting tips | No |
| Text4baby | Apple App Store | Voxiva | Health & Fitness | 47.9 | iOS 8.0 or later. | Vaccine tracking, general development advice, general parenting advice | No |
| Tryde | Apple App Store | RCJJRB LLC | Education | 17.9 | iOS 9.0 or later. | Development screening | No |
| Name of App | App Store | Developer | Category | Size (in MB) | Operating systems supported | Primary features in app | Has in-app advertisements? |
| Vroom | Apple App Store | Bezos Family Foundation | Education | 30.7 | iOS 11.0 or later. | Activities to support development | No |
| webmd baby | Apple App Store | WebMD Health Corporation | Health & Fitness | 65.7 | iOS 10.0 or later. | Tracking feeding, diaper, sleep; random tips for parenting by week (up to 2 years old) | No |
| wunder - baby tracker | Apple App Store | Oya Inc | Education | 82.4 | iOS 12.3 or later. | Development screening | No |
| 100 Baby Growth, Infant Care & Parenting Facts | Google Play Store | TipsBook | Parenting | 16.7 | Android 4.1 and up | Quiz/Trivia about child health | Yes |
| 50 BAby & Infant Care Quiz; for new Parents | Google Play Store | Anish Nrk | Parenting | 7.2 | Android 4.0.3 and up | Quiz/Trivia about child health | Yes |
| baby + - your baby tracker | Google Play Store | Philips Consumer Lifestyle B.V. (partnership with Philips Avent) | Parenting | 126 | Android 5.0 and up | Tracking feeding, diaper, sleep, Provides articles about developmental milestones and timelines | Yes |
| baby app, baby tracker | Google Play Store | Softmint | Parenting | 1.6 | Android 4.0.3 and up | Tracking feeding, diaper, sleep | No |
| Name of App | App Store | Developer | Category | Size (in MB) | Operating systems supported | Primary features in app | Has in-app advertisements? |
| baby breastfeeding tracker | Google Play Store | Sevenlogics, INC | Parenting | 25 | Android 5.0 and up | Tracking feeding, diaper, sleep | Yes |
| Baby Care - Feeding timer & Daily Baby Log | Google Play Store | Yalintech | Parenting | 12 | Android 5.1 and up | Tracking feeding, diaper, sleep | Yes |
| Baby Care - Newborn Feeding, Diaper, Sleep Tracker | Google Play Store | Hightech Solution | Parenting | 11 | Android 5.0 and up | Tracking feeding, diaper, sleep | Yes |
| baby care - track baby growth | Google Play Store | Breet.Jia | Medical | 9.8 | Android 4.1 and up | Tracking feeding, diaper, sleep | No |
| baby care log & tracker | Google Play Store | Steveloper | Parenting | 25 | Android 4.4 and up | Tracking feeding, diaper, sleep | Yes |
| Baby Care Parent Guide: Birth-Newborn Development | Google Play Store | Yoanna Tech | Parenting | 2.6 | Android 3.0 and up | Article reading (website embedded into app) about basics of taking care of child up to age 1 | Yes |
| Baby Care Tracker - Breastfeeding | Google Play Store | digerati.cz | Parenting | 10 | Android 4.4 and up | Tracking feeding, diaper, sleep | Yes |
| baby care week by week. tips | Google Play Store | kukipukie | Books & Reference | 9.6 | Android 5.0 and up | Articles about health and development up to age 3 | Yes |
| Name of App | App Store | Developer | Category | Size (in MB) | Operating systems supported | Primary features in app | Has in-app advertisements? |
| baby daybook - newborn breastfeeding tracker app | Google Play Store | Baltapis | Parenting | 22 | Android 4.1 and up | Tracking feeding, diaper, sleep | No |
| baby development - growth log | Google Play Store | Steveloper | Medical | 6 | Android 41. and up | Tracking child weight and height only | Yes |
| Baby Development Guide | Google Play Store | Pregnancy and Baby Apps | Health & Fitness | 4.6 | Android 4.0 and up | View online "free forum" in an app, paired with articles | Yes |
| Baby Development Milestone: Week by Week | Google Play Store | zayn media | Parenting | 5.6 | Android 4.4 and up | Articles about developmental milestones and taking care of chid up to 5 years old | Yes |
| Baby Diary - Feeding, Sleep and Healthy tracker | Google Play Store | GTSStar | Parenting | 11 | Android 6.0 and up | Tracking feeding, diaper, sleep | Yes |
| Baby Feeding Tracker - Newborn Feeding and Care | Google Play Store | Rsky | Parenting | 5.7 | Android 5.0 and up | Tracking feeding, diaper, sleep | Yes |
| Name of App | App Store | Developer | Category | Size (in MB) | Operating systems supported | Primary features in app | Has in-app advertisements? |
| baby food chart | Google Play Store | Definicija hrane | Parenting | 45 | Android 4.4 and up | Tracking feeding, diaper, sleep | Yes |
| Baby Grow: feeding, sleep, diaper, expense tracker | Google Play Store | Apponance, Inc. | Parenting | 36 | Android 5.0 and up | Tracking feeding, diaper, sleep | Yes |
| Baby Growth | Google Play Store | OWN-IT | Parenting | 9.5 | Android 5.1 and up | Quiz/Trivia about child health | No |
| Baby Growth & Development | Google Play Store | Dr Deepak Choudhury | Medical | 3.9 | Android 4.2 and up | Development screening | Yes |
| baby growth calculator | Google Play Store | Trusted Assets | Parenting | 6.3 | Android 4.0 and up | Development screening | Yes |
| baby growth month by month | Google Play Store | ARUNAS APPS LLP | Parenting | 3.9 | Android 4.1 and up | Development screening | Yes |
| Baby Info - Baby Tracker for feeds, sleep and more | Google Play Store | MartAndTrep | Parenting | 6.2 | Android 4.4 and up | Tracking feeding, diaper, sleep | Yes |
| baby journal: child growth, milestone book & diary | Google Play Store | Master App Solutions | Parenting | 15 | Android 4.1 and up | Tracking feeding, diaper, sleep, sentimental milestones | No |
| Name of App | App Store | Developer | Category | Size (in MB) | Operating systems supported | Primary features in app | Has in-app advertisements? |
| baby language: talking baby | Google Play Store | Pranata House & Gallery | Education | Varies with device | Android 5.0 and up | Information about 5 different baby sounds | No |
| baby log (stash, nurse, growth, sleep, feed) | Google Play Store | Compass Apps | Parenting | 8.6 | Android 6.0 and up | Tracking feeding, diaper, sleep | Yes |
| Baby Major Steps | Google Play Store | e-verbum | Parenting | 4.2 | Android 4.1 and up | Development screening | Yes |
| Baby Manager - Breastfeeding Log and Tracker | Google Play Store | LiveKid | Parenting | 23 | Android 4.3 and up | Tracking feeding, diaper, sleep | Yes |
| baby skills | Google Play Store | BabySkills | Lifestyle | 4.4 | Android 2.3 and up | Development screening | Yes |
| Baby Tracker - Feeding, Diaper, Activity, Sleep | Google Play Store | 18K Development | Parenting | 26 | Android 5.0 and up | Tracking feeding, diaper, sleep | Yes |
| Baby Tracker - Newborn Care from Head to Toe | Google Play Store | Doğan Bilişim ve İnternet Teknolojileri | Parenting | 63 | Android 5.0 and up | Information about parenting/milestones, sounds to soothe baby, games to play, blog with info about baby, growth percentile calculator, vaccine tracker | Yes |
| Name of App | App Store | Developer | Category | Size (in MB) | Operating systems supported | Primary features in app | Has in-app advertisements? |
| Baby Tracker - Newborn Feeding, Diaper, Sleep Log | Google Play Store | NIGHP SOFTWARE | Parenting | 19 | Android 4.4 and up | Tracking feeding, diaper, sleep | Yes |
| Baby tracker - newborn log (blue icon) | Google Play Store | Stay Fit With Samantha | Parenting | 3.4 | Android 4.4 and up | Tracking feeding, diaper, sleep | Yes |
| baby tracker - newborn tracker | Google Play Store | wing wing | Parenting | 24 | Android 4.1 and up | Tracking feeding, diaper, sleep | Yes |
| baby tracker - sleep, breastfeeding, food, diaper | Google Play Store | Baby Tracker App | Parenting | 11 | Android 5.0 and up | Tracking feeding, diaper, sleep | Yes |
| Baby Tracker:Feeding,Diaper,Sleep for Newborn | Google Play Store | PM Apps 2020 | Tools | 6.3 | Android 4.4 and up | Tracking feeding, diaper, sleep, and random parenting tips | Yes |
| Baby Tracker. Breastfeeding Log & Nursing - MeGrow | Google Play Store | Myamplifiers.com | Parenting | 9.7 | Android 4.1 and up | Tracking feeding, diaper, sleep | No |
| Name of App | App Store | Developer | Category | Size (in MB) | Operating systems supported | Primary features in app | Has in-app advertisements? |
| Baby Words - speech and language development diary | Google Play Store | BigMeApps | Parenting | 3 | Android 6.0 and up | Tracking baby vocabulary/word development | Yes |
| Baby's Brain Development week by week | Google Play Store | MasterpieceApps | Health & Fitness | 6.6 | Android 4.1 and up | Information about child health and parenting tips | Yes |
| BabyAppy: formula feeding, sleep and diapers | Google Play Store | BigMeApps | Parenting | 4.2 | Android 4.4 and up | Tracking feeding, diaper, sleep | Yes |
| babybeats early intervention resource | Google Play Store | Advanced Bionics LLC | Education | 16 | Android 5.0 and up | Music and movement for babies with hearing loss | No |
| Babygogo PArenting - Baby Care & Pregnancy Tips | Google Play Store | Babygogo | Parenting | 7.3 | Android 4.1 and up | Parenting tips via forums/feed/SNS/connect with doctors/shopping | No |
| babygym | Google Play Store | MotorikApp ApS | Parenting | 40 | Android 5.0 and up | Activities to support physical/motor development and connection with parents | No |
| Name of App | App Store | Developer | Category | Size (in MB) | Operating systems supported | Primary features in app | Has in-app advertisements? |
| Babylog (Parenting, Track & Analysis) | Google Play Store | ForestApps | Parenting | 6.2 | Android 4.3 and up | Quiz/Trivia about child health | Yes |
| bee parenting | Google Play Store | Early Childhood Development Pvt Ltd. | Parenting | 56 | Android 5.0 and up | Tracking feeding, diaper, sleep | No |
| beurer babycare | Google Play Store | Beurer GmbH | Parenting | 22 | Android 5.0 and up | Tracking feeding, diaper, sleep | No |
| Boky - Baby Diary | Google Play Store | Cuberob | Parenting | 10 | Android 4.4 and up | Tracking feeding, diaper, sleep | No |
| bonbaby - baby health tracker | Google Play Store | FEMOMETER LIMITED | Parenting | 22 | Android 4.3 and up | Monitor baby fever, weight, height, medications | No |
| Best App for Pregnancy Tracker & Baby Care | Google Play Store | Amit Kulhari | Parenting | 21 | Android 5.0 and up | Tracking feeding, diaper, sleep | Yes |
| Breastfeeding Newborn tracker, pump and baby diary | Google Play Store | Whisper Arts | Parenting | 14 | Android 5.0 and up | Tracking feeding, diaper, sleep | Yes |
| Name of App | App Store | Developer | Category | Size (in MB) | Operating systems supported | Primary features in app | Has in-app advertisements? |
| child development - wachanga | Google Play Store | Wachanga | Parenting | 29 | Android 5.0 and up | Milestone diary, weekly development tips | No |
| Child Development 0 to 6 | Google Play Store | Tibus | Education | 6.8 | Android 4.1 and up | Quiz/Trivia about child health | No |
| Child Development (hartwell) | Google Play Store | harwell mhunduru | Health & Fitness | 7.2 | Android 5.0 and up | Development screening | No |
| Child Development Milestones | Google Play Store | Academy for Professional Excellence | Books & Reference | 2.8 | Android 4.1 and up | Information about development and milestones (list with reading) | No |
| Child Development | Google Play Store | Mapri apps | Medical | 2.7 | Android 2.3.3 and up | Tracking feeding, diaper, sleep | No |
| Child Growth Diary | Google Play Store | CosTheta | Health & Fitness | 2.9 | Android 5.0 and up | Development screening | No |
| Child Growth Tracking | Google Play Store | EXRL | Parenting | 21 | Android 5.0 and up | Tracking head, body, weight against WHO percentiles | Yes |
| child learning development | Google Play Store | Success Freedom System | Education | 17 | Android 5.0 and up | Articles about parenting advice | Yes |
| Name of App | App Store | Developer | Category | Size (in MB) | Operating systems supported | Primary features in app | Has in-app advertisements? |
| Continua Kids - Child Growth & Development | Google Play Store | Wayu Health | Medical | 46 | Android 5.0 and up | Development screening | No |
| your baby month by month | Google Play Store | devalip | Parenting | 7.1 | Android 4.1 and up | Information about child development by month | Yes |
| womanlog baby calendar | Google Play Store | Pro Active App SIA | Health & Fitness | 21 | Android 4.1 and up | Tracking feeding, diaper, sleep | Yes |
| Turalura Baby: Track Growth Percentile & Milestone | Google Play Store | KosmicDust | Parenting | 23 | Android 5.0 and up | Development screening | Yes |
| Talli Baby | Google Play Store | Babylogger, LLC | Medical | 38 | Android 5.0 and up | Tracking feeding, diaper, sleep | No |
| SuperMama: Breast Feeding and Baby Daybook App | Google Play Store | Lighthouse GmbH | Parenting | 57 | Android 6.0 and up | Tracking feeding, diaper, sleep | No |
| Playfully Baby Development Activities & Milestones | Google Play Store | Playfully | Parenting | 9.9 | Android 4.4 and up | Milestone tracking and activities to support those milestones | No |
| Baby Tracker, Breastfeeding, Diapering: ParentLove | Google Play Store | Baby Tracker by Coquisoft | Parenting | Varies with device | Android 4.4 and up | Tracking feeding, diaper, sleep | Yes |
| Name of App | App Store | Developer | Category | Size (in MB) | Operating systems supported | Primary features in app | Has in-app advertisements? |
| Parent Sense: Activity & Milestones Baby Tracker | Google Play Store | Sense-IT Limited | Parenting | 35 | Android 5.0 and up | Tracking feeding, diaper, sleep | No |
| ovia parenting: baby tracker, breastfeeding timer | Google Play Store | Ovia Health | Medical | 21 | Android 6.0 and up | Tracking feeding, diaper, sleep; Tracking/supporting maternal wellness, developmental milestone checklist, activities to support development (in article form) | Yes |
| OBAby | Google Play Store | Overlake Medical Center & Clinics | Health & Fitness | 30 | Android 4.4 and up | Articles about newborn/pregnancy (customizable) care and tracking feeding, diaper, sleep | No |
| Daily Baby Tracker | Google Play Store | Ugurcan Ozkan | Parenting | 8.3 | Android 5.0 and up | Tracking feeding, diaper, sleep | Yes |
| child growth tracker (crawling baby) | Google Play Store | ABQ App Source, LLC | Medical | 8.7 | Android 4.1 and up | Tracking feeding, diaper, sleep | Yes |
| Nuttri - Baby Food: Guide to starting solids | Google Play Store | MultiMension Inc. | Parenting | 20 | Android 4.4 and up | Meal Planner for Child (transition to solid foods) | Yes |
| Name of App | App Store | Developer | Category | Size (in MB) | Operating systems supported | Primary features in app | Has in-app advertisements? |
| Nod - Baby Sleep Coach & Feeding Tracker | Google Play Store | Rest Devices, Inc. | Parenting | 19 | Android 4.1 and up | Sleep Coaching/Tracking and Feeding Tracker | Yes |
| Newborn & Baby Development Guide | Google Play Store | Parenting Pets Care Tips | Parenting | 4 | Android 4.1 and up | Quiz/Trivia about child health | Yes |
| MyPreemie app | Google Play Store | Graham's Foundation | Medical | 24 | Android 4.4 and up | Growth tracking for premature baby, Parenting advice for premature baby | No |
| guide to newborn care | Google Play Store | Saleha Group | Parenting | 4.4 | Android 4.1 and up | Articles about caring for newborn | Yes |
| hatch baby - activity tracker | Google Play Store | Hatch Sleep | Health & Fitness | 51 | Android 4.4 and up | Tracking feeding, diaper, sleep | No |
| Immunization Planner | Google Play Store | NiveshNiti | Parenting | 12 | Android 4.0 and up | Tracking Immunizations and Developmental Milestone Checklist | Yes |
| Indian PRegnancy Advice, Baby Care, Parenting Tips | Google Play Store | Parentune - Parenting, Child care Growth Tracker | Parenting | 13 | Android 5.1 and up | Parenting advice forum (ask doctors/experts, connect with other parents, read articles, chat) | No |
| Name of App | App Store | Developer | Category | Size (in MB) | Operating systems supported | Primary features in app | Has in-app advertisements? |
| Indigo. Smart activities. | Google Play Store | baby development app | Parenting | 8.9 | Android 4.4 and up | Development screening | No |
| Kiduga: BAby Development | Google Play Store | Kiduga Digital | Parenting | 8.1 | Android 7.0 and up | Tracking feeding, diaper, sleep | No |
| kidzgrow child development app | Google Play Store | KidzGrow Aps Pte Ltd | Education | 7.3 | Android 4.0 and up | Development screening | Yes |
| KinderPass: Baby Development, Activities & Classes | Google Play Store | KinerPass Pte Ltd. | Education | 54 | Android 5.0 and up | Developmental milestones checklist, general parenting advice articles, activities to support baby development | No |
| Little Ones tm | Google Play Store | LittleONES | Education | 12 | Android 4.4 and up | Parenting coaching programs, online articles, open forums for parents to communicate | No |
| mediclinic baby - baby | Google Play Store | Mediclinic (Pty) Ltd | Medical | 35 | Android 4.1 and up | Focus on pregnancy ddevelopment with month by month information about milestones and development (short paragraph) | No |
| Name of App | App Store | Developer | Category | Size (in MB) | Operating systems supported | Primary features in app | Has in-app advertisements? |
| Mom And Baby - Love Baby 3000 | Google Play Store | RemifasonPR | Parenting | 5.4 | Android 4.4 and up | Tracking feeding, diaper, sleep | No |
| mother & baby care | Google Play Store | ALAA Apps | Parenting | 16 | Android 5.0 and up | Random parenting tips articles | Yes |
| my baby pediatric growth | Google Play Store | Sistema Informaticos | Health & Fitness | 4.1 | Android 4.2 and up | Percentiles growth tracker (weight and height), recipes, first foods | Yes |
| my baby: development tracker app 0-12 | Google Play Store | Hylal Health Apps | Parenting | 3.7 | Android 7.0 and up | Weight and Height Tracker, sentimental photo album, general articles about parenting/activities/tips | No |
| development of the child up to a year | Google Play Store | identdevelop | Parenting | 15 | Android 4.1 and up | Articles about parenting and child development | Yes |
| early child development kit guide | Google Play Store | Grow Together | Parenting | 7.5 | Android 4.1 and up | Random activities to do with child (not based on age) | Yes |
| Erby Breastfeeding tracker, pump log & baby diary | Google Play Store | Whisper Arts | Parenting | 20 | Android 5.0 and up | Tracking feeding, diaper, sleep | Yes |
| Name of App | App Store | Developer | Category | Size (in MB) | Operating systems supported | Primary features in app | Has in-app advertisements? |
| Feed Baby - Baby Tracker | Google Play Store | Penguin Apps | Parenting | 19 | Android 4.1 and up | Tracking feeding, diaper, sleep | Yes |
| GLOW. Baby Tracker & Feeding, Diaper, Sleep Log | Google Play Store | Glow Inc | Parenting | 34 | Android 5.0 and up | Tracking feeding, diaper, sleep | Yes |
| Growth Chart, Development Milestones & Vaccination | Google Play Store | Growth Book | Parenting | 10 | Android 5.0 and up | Trackng growth, development, food, vaccines, online consultation with provider | No |
