# Supplementary material for: Quality of Mobile Apps for Child Development Support: Search in App Stores and Content Analysis
Source: JMIR Pediatr Parent. 2022 Nov 8;5(4):e38793. doi: 10.2196/38793 (PMC9682452; doi:10.2196/38793)

## Multimedia Appendix 2

### Section A: Search Strings by App Store

| App Store | Search String |
| --- | --- |
| Apple App Store | Baby+health  Baby+development  Baby+milestone  Baby+tracking  Child+development  Child+milestone  Child+tracking  Child+health |
| Google Play Store | Baby+health  Baby+development  Baby+milestone  Baby+tracking  Child+development  Child+milestone  Child+tracking  Child+health |

### Section B: Apps Excluded in each step of the screening process


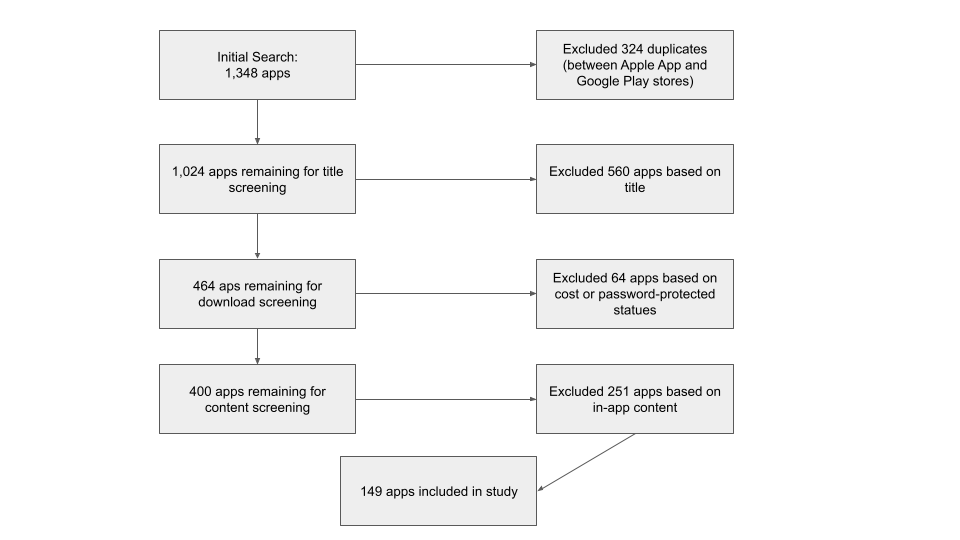

Supplement: Multimedia Appendix 2 [file pediatrics_v5i4e38793_app2.docx]
